# Role of Chemical Reduction and Formulation of Graphene Oxide on Its Cytotoxicity towards Human Epithelial Bronchial Cells

**DOI:** 10.3390/nano13152189

**Published:** 2023-07-27

**Authors:** Marco Pelin, Clara Passerino, Adriana Rodríguez-Garraus, Michela Carlin, Silvio Sosa, Satu Suhonen, Gerard Vales, Beatriz Alonso, Amaia Zurutuza, Julia Catalán, Aurelia Tubaro

**Affiliations:** 1 Department of Life Sciences, University of Trieste, Via Fleming 22, 34127 Trieste, Italy; clara.passerino@gmail.com (C.P.); michela.carlin@phd.units.it (M.C.); ssosa@units.it (S.S.); tubaro@units.it (A.T.); 2 Finnish Institute of Occupational Health, Box 40, Työterveyslaitos, 00032 Helsinki, Finland; adriana.rodriguezgarraus@ttl.fi (A.R.-G.); satu.suhonen@ttl.fi (S.S.); gerard.valessegura@ttl.fi (G.V.); julia.catalan@ttl.fi (J.C.); 3 Graphenea S.A., Mikeletegi 83, 20009 San Sebastián, Spain; b.alonso@graphenea.com (B.A.); a.zurutuza@graphenea.com (A.Z.); 4 Department of Anatomy Embryology and Genetics, University of Zaragoza, 50013 Zaragoza, Spain

**Keywords:** graphene-based materials, inhalational exposure, occupational exposure, physicochemical properties, graphene oxide, reduced graphene oxide, cytotoxicity, safety assessment, hazard characterization

## Abstract

Graphene-based materials may pose a potential risk for human health due to occupational exposure, mainly by inhalation. This study was carried out on bronchial epithelial 16HBE14o− cells to evaluate the role of chemical reduction and formulation of graphene oxide (GO) on its cytotoxic potential. To this end, the effects of GO were compared to its chemically reduced form (rGO) and its stable water dispersion (wdGO), by means of cell viability reduction, reactive oxygen species (ROS) generation, pro-inflammatory mediators release and genotoxicity. These materials induced a concentration-dependent cell viability reduction with the following potency rank: rGO > GO >> wdGO. After 24 h exposure, rGO reduced cell viability with an EC_50_ of 4.8 μg/mL (eight-fold lower than that of GO) and was the most potent material in inducing ROS generation, in contrast to wdGO. Cytokines release and genotoxicity (DNA damage and micronucleus induction) appeared low for all the materials, with wdGO showing the lowest effect, especially for the former. These results suggest a key role for GO reduction in increasing GO cytotoxic potential, probably due to material structure alterations resulting from the reduction process. In contrast, GO formulated in a stable dispersion seems to be the lowest cytotoxic material, presumably due to its lower cellular internalization and damaging capacity.

## 1. Introduction

Graphene is a two-dimensional material consisting of a monolayer of sp^2^-bonded carbon atoms arranged in a honeycomb lattice. Since its isolation in 2004 by mechanical exfoliation of highly oriented pyrolytic graphite, it has become a hotly debated topic in the fields of material science, nanotechnology, physics and chemistry, among others [1,2]. The interest in graphene and graphene-based materials (GBMs) has been progressively increasing over the years, due to their unique physicochemical properties. These materials are characterized by large surface area, very high mechanical strength and thermal and electrical conductivity [3,4,5]. The family of GBMs includes few-layer graphene (FLG), its highly oxidized form graphene oxide (GO), its reduced form (rGO) and other variously functionalized graphene materials [6]. GO—a material rich in oxygen-containing functional groups, such as hydroxyl, carbonyl, carboxyl and epoxy groups, which include the element oxygen (O)—can be obtained by oxidation of graphite under highly acidic conditions [7,8,9] and, subsequently, potentially reduced, either chemically or thermally, to obtain rGO [10]. Among this multitude of GBMs, GO is certainly considered the most promising for its biological applications, due to its good dispersibility in biological media with respect to other materials, and its ease of functionalization [11].

For a clear classification of this plethora of materials, three physicochemical descriptors can be considered: the carbon-to-oxygen (C/O) atomic ratio, the average lateral size and the number of graphene layers [12]. However, it should be noted that GBM biological effects, including their toxic potential, may be influenced by each different physicochemical property [13]. For instance, an in vitro study on human keratinocytes exposed to a panel of different GBMs demonstrated that the oxidation state (index of the content of oxygen-containing functional groups in the material) is the main physicochemical property influencing GBM cytotoxicity, with GO being more potent than FLG [14]. A flake-size dependent cytotoxicity of GO was also observed on human lung adenocarcinoma cells exposed to three GO characterized by different dimensions (size range: 150–1000 nm), the largest sized GO resulting in lower cytotoxicity, while the smallest sized GO showed the highest toxicity in terms of cell viability reduction [15]. An in vitro study on murine DC2.4 dendritic cells concluded that the effect of GO on cell viability is also closely related to the number of layers, with the mono-layer GO being less potent in reducing cell viability than the multi-layer GO [16].

Over time, GBM physicochemical characteristics have been studied, deepened, and improved, with the aim of expanding their applications and progressively enabling a more widespread practical use. However, there are many unanswered questions concerning their effects on human health and the environment, thus requiring further toxicity studies to characterize the general safety of these materials, as well as in relation to their physicochemical properties specifically [13]. The major risk to human health associated with GBM exposure is correlated to occupational exposure, during both industrial and laboratory scale production. In these scenarios, exposure to GBMs can occur mainly by inhalation, cutaneous and ocular routes and, less frequently, by ingestion [17]. Inhalation is the main method of exposure and also the most studied route, both in vivo and in vitro, as previously summarized in various reviews [13,17,18,19]. To assess pulmonary toxicity, in vivo studies can be performed after exposure to GBMs by inhalation, pharyngeal aspiration or intratracheal instillation [20]. Regarding the effects by inhalation of graphene or graphene nanoplatelets (6 h, 1 week, 4 weeks exposure), no significant changes in the body and organ weights were observed in rats; however, graphene pulmonary persistence after phagocytosis by alveolar macrophages was recorded [21,22]. In addition, changes in the bronchoalveolar lavage fluid composition in terms of cytology parameters, inflammatory mediators and enzyme activity were found [23]. In vitro data on GBM toxic effects towards several cell types, such as different kinds of human lung cells and co-culture models, consisting of bronchial epithelial cells (16HBE) and monocytes (THP-1), among others, are available. For instance, an in vitro study on human lung fibroblasts exposed to GO (1–100 µg/mL), for 2 h and up to 24 h, demonstrated a concentration-dependent reduction of cell viability, significant at concentrations equal or higher than 50 μg/mL at all exposure times. This cytotoxicity appeared to be caused by oxidative stress, as GO induced significant concentration-dependent production of reactive oxygen species (ROS) [24]. The oxidative stress-mediated cytotoxicity of three graphene derivates was highlighted in a subsequent study on bronchial epithelial cells (BEAS-2B) and alveolar epithelial cells (A549), identifying an inverse correlation between viability reduction and increased ROS generation [25]. Pro-inflammatory response in terms of IL-8 expression in A549 cells was assessed after 6 and 24 h exposure to GO or rGO (10–160 µg/mL), which induced a significant IL-8 increase after 6 h exposure, at concentrations of 40 and 160 µg/mL, respectively [26].

Regarding the genotoxic effects of GO and its derivatives, several materials were evaluated for their effects towards BEAS-2B cells using the comet assay, which showed differences in the potency of double strand breaks induction after 24 h exposure to 10–50 µg/mL. In general, pristine and functionalized graphene nanoplatelets were found to be more potent genotoxicans than single-layer or few-layers GO [27]. Hinzman and colleagues also observed a differential genotoxic effect, depending on GBM modifications. Even though they are not pulmonary cells, in U87 cells, DNA damage was induced by pristine graphene, rGO and graphite, while no effect was observed for GO in the same system [28].

Hence, considering the potential adverse effects of GO on pulmonary cells, this study was carried out in vitro on 16HBE14o− bronchial epithelial cells. The literature data on GO often show discordant results, frequently associated with different toxic potencies depending on the source, nature, preparation and physicochemical properties of the tested GO; therefore, our study aimed to investigate the role of two important features of GO, potentially affecting its safety profile: (i) its chemical reduction and (ii) its different formulation as powder or in a stable water dispersion. To this end, GO prepared as a powder or in a stable dispersion form (wdGO), and its chemically reduced form (rGO), were evaluated for their effects on 16HBE14o− bronchial epithelial cells in terms of cell viability reduction, ROS production, the release of pro-inflammatory mediators and genotoxic potential though a comparative approach.

## 2. Materials and Methods

### 2.1. Materials

GO, rGO and stable water dispersion of GO (wdGO), together with their physicochemical characterization, were provided by Graphenea S.A. (San Sebastian, Spain). Briefly, GO was prepared by a modified Hammers’ method (patent n. EP3070053B1). rGO was prepared by chemical reduction of GO using ascorbic acid (patent n. WO2019145378A1) and wdGO was prepared by subjecting GO to a dilution and to an ultrasound process. Each material was physicochemically characterized by elemental analysis, X-ray diffraction (XRD), thermogravimetric analysis (TGA) and transmission electron microscopy (TEM).

Each material was dispersed in 0.1% bovine serum album (BSA) solution to achieve a stable dispersion, allowing cells treatment as previously reported [29]. Dispersions were sonicated (44 ± 2 kHz) for 45 min, followed by a vortexing step every 15 min. Once dispersed, pH was slightly acid (pH values between 6 and 7), but once diluted in cell culture media (see below) at the highest concentration for cell treatment (100 µg/mL), pH values were between 7.5 and 7.7. The stability of each material dispersion (1 mg/mL) in 0.1% BSA was assessed by UV-visible spectrophotometric analysis, measuring absorbance at 660 nm for up to 24 h.

### 2.2. Physicochemical Characterization

Elemental analysis was performed through the *FlashSmart CHNS/O* (ThermoFisher, Madrid, Spain) directly on the powder materials. For wdGO, samples were prepared by casting 10 mL of 4 mg/mL dispersion in a 4 cm diameter mold. The analysis was performed after drying overnight in a vacuum oven at 60 °C.

TGA analysis was performed using a TA Instruments Discovery TGA550. The dried samples were analyzed up to 1000 °C in N_2_ atmosphere with a 10 °C/min ramp.

XRD was carried out using a Rigaku Miniflex 600. The dried samples were analyzed at 40 Kv and 15 mA with a Cu tube from 7 to 70 °C.

TEM analysis was carried out to determine average lateral dimensions of the three materials. Dispersions of GO, wdGO and rGO in cell culture media (Minimum Essential Medium, MEM) (Sigma Aldrich; Milan, Italy) were properly diluted and dropped on a lacey copper grid (100 micron, 300 mesh, coated with carbon film) and dried at room temperature to remove the solvent. Samples were investigated using a Philips EM208 TEM and RADIUS 2.0 software (EMSIS GmbH; Muenster, Germany). Lateral dimensions distributions were analyzed offline using the ImageJ software.

### 2.3. Tumor Necrosis Factor (TNF)-α Expression Test (TET Assay) 

Endotoxin contamination of GBMs was assessed by a modified version of the TET assay previously described [30], using macrophages obtained by differentiation of human THP-1 monocytes. Briefly, THP-1 cells, differentiated using 50 nM phorbol-12-myristate-13-acetate (PMA) for 24 h, were subsequently exposed to a non-cytotoxic concentration (1 µg/mL) of each GBM for 24 h, in the presence or absence of polymyxin B sulfate (10 µM). Macrophages were also exposed to different concentrations of lipopolysaccharide (LPS; 0.01–100 ng/mL) to generate a standard curve based on TNF-α cell release; 100 ng/mL LPS was included as a positive control. Afterward, supernatants were collected and TNF-α was quantified with a specific ELISA kit, following the manufacturer’s instructions (Diaclone; Tema Ricerca, Bologna, Italy). LPS was quantified in each GBM on the basis of a standard curve generated on LPS-induced TNF-α release data. Data are reported as the mean LPS concentration (EU/mL) ± SE in each sample.

### 2.4. Cell Culture 

The human bronchial epithelial 16HBE14o− cell line was provided by Dr. Dieter Gruenert (University of California; San Francisco, CA, USA). Cells were maintained in MEM supplemented with 10% fetal bovine serum (FBS), 200 mM L-glutamine, 100 IU/mL penicillin and 100 µg/mL streptomycin (Euroclone; Milan, Italy). Cell passage was performed once a week and cell cultures were maintained in a humidified incubator at 37 °C with 5% CO_2_. For the evaluation of GBM cytotoxicity, cells were seeded in 96-well plates at a density of 1 × 10^4^ cells/well.

THP-1 cells, used for TET assay, were cultured in RPMI-1640 supplemented with 10% FBS, 1% glutamine, 100 µg/mL penicillin/streptomycin at 37 °C under a humidified 95% air/5% CO_2_ atmosphere.

### 2.5. May-Grunwald Giemsa Staining

To evaluate cell morphology, cells (5 × 10^4^/well) were seeded in a 24-well plate for 2 days. Cells were then exposed to GBMs (10 μg/mL) for 24 h, washed two times with phosphate buffered saline (PBS) for 10 min each on an orbital shaker (75 rpm) and fixed for 1 min in MeOH. Cells were then stained for 5 min in May–Grunwald stain (0.25% in MeOH) and twice for 7 min in Giemsa stain solution (0.1% in PBS). Cells were then washed with distilled water two times for 10 min each on an orbital shaker (75 rpm), and images were taken by an inverted light Leica DMi1 microscope, equipped with a FLEXACAM C1 standard camera (Leica Microsystems; Milan, Italy) at a 40× magnification.

### 2.6. Confocal Microscopy

Cell internalization of GO derivatives was assessed by laser confocal microscopy. A total of 6 × 10^4^ 16HBE14o− cells were seeded onto clean 16-mm ø coverslips (VWR international; Radnor, PA, USA), which were placed onto 12-well plates (Corning Inc.; Corning, NY, USA) for 24 h. Afterwards, cells were exposed to 25 µg/mL of each material for 24 h. Then, cells were stained in the dark, using CellMask™ Deep Red plasma membrane stain solution (1:1000, 5 µg/mL, in DPBS, 37 °C, 10 min; Thermofisher Scientific; Waltham, MA, USA), fixed in 4% formaldehyde in PBS (37 °C, 10 min) and stained using Hoechst 33342 nuclei staining solution (5 µg/mL in PBS, 37 °C, 10 min; Invitrogen; Waltham, MA, USA). Cells were washed three times in PBS after each step. Then, the coverslips were mounted on super frost slides (VWR international; Radnor, PA, USA) using Vectashield (Vector Laboratories Inc.; Burlingame, CA, USA) and sealed with nail polish.

Images were obtained by a Carl Zeiss LSM 510 confocal laser scanning microscope (Zeiss; Oberkochen, Germany) using the 405 nm (Hoechst 33342 staining, nuclei), 543 nm (graphene material) and 630 nm (CellMask™ staining, membrane) lasers and a 40× magnification objective. The graphene materials were observed due to their own light reflective properties. Confocal images were obtained and processed using the Zen 2008 software (Zeiss; Oberkochen, Germany).

### 2.7. WST-8 Reduction Assay

The effect of GBMs on 16HBE14o− cells viability was assessed by the WST-8 assay using the Cell Counting Kit (CCK)-8 (Sigma Aldrich; Milan, Italy), following the manufacturer’s instructions. After exposure to GBMs (0.4–100.0 μg/mL), cells were washed two times with PBS (200 μL/well) and incubated for 4 h with fresh medium (100 μL/well) containing 10 μL of WST-8 reagent. Absorbance was subsequently read at 450 nm using the automated microplate reader FLUOstar Omega (BMG LABTECH, Germany). Data are the mean ± SE of at least 3 independent experiments performed in triplicate and reported as % of cell viability in cells exposed to GBMs with respect to untreated control cells (negative control).

### 2.8. Dichlorofluorescin Diacetate (DCFDA) Assay

ROS production induced by GO, rGO or wdGO was evaluated by the 2′,7′-dichlorofluorescin diacetate (DCFDA) assay, as previously described [31]. Before cell treatment, cells were incubated with medium (100 μL/well) containing 100 μM DCFDA for 30 min, at 37 °C in the dark. Then, the cells were washed twice with PBS added with Ca^2+^ and Mg^2+^ (200 μL/well) and exposed to GBMs (0.4–100.0 μg/mL) in complete medium without phenol red. As a positive control, 1 mM 2,2′-Azobis(2-methylpropionamidine) dihydrochloride (AAPH; Sigma Aldrich; Milan, Italy), a free radical initiator, was used. The fluorescence was read by the microplate fluorometer FLUOstar Omega (BMG LABTECH; Germany), at an excitation wavelength of 485 nm and an emission wavelength of 520 nm, immediately after the exposure to GBMs and after increasing exposure times (45 min–24 h). Data are the mean ± SE of at least 3 independent experiments performed in triplicate, and are reported as % of ROS increase with respect to negative control (cells not exposed to GBMs).

### 2.9. Pro-Inflammatory Mediators Analysis

The pro-inflammatory response of 16HBE14o− cells exposed to GO, rGO or wdGO (10 μg/mL) was evaluated using specific enzyme-linked immunosorbent assays (ELISA). 16HBE14o− cells were seeded in T25 cell culture flasks at the density of 10 × 10^5^ cells/flask and incubated for 48 h at 37 °C and 5% CO_2_. Subsequently, cells were exposed to 10 μg/mL of each GBM for 24 h. As positive control, cells were exposed to 1 μg/mL LPS for 24 h; 1 μg/mL MITSUI-7 Multiwall Carbon-Nanotubes (MWCNT) were used as reference material. After treatment, supernatants were collected and the following pro-inflammatory mediators were quantified: interleukin (IL)-1α, IL-1β, IL-6, IL-8, IL-18, TNF-α, monocyte chemoattractant protein-1 (MCP-1), prostaglandin E_2_ (PG-E_2_), interferon-gamma (INF-γ), granulocyte-macrophage colony-stimulating factor (GM-CSF), eosinophil chemotactic factor (ECF/CCL11), regulated upon activation normal T cell expressed and secreted (RANTES). Each mediator was quantified using a specific ELISA kit, following the manufacturer’s instructions; ELISA kits were provided by Diaclone (Tema Ricerca; Bologna, Italy) and by Elabscience (Milan, Italy). Data are reported as pg/mL of each mediator released in the media and are the mean ± SE of three independent experiments.

### 2.10. Genotoxicity Analysis

The concentrations of GBMs tested in the genotoxicity assays were set based on a preliminary cytotoxicity assay, as recommended by the OECD TG 487 [32]. Cytotoxicity was assessed using the CellTiter-GloVR Luminescent Cell Viability Assay (Promega; Madison, WI, USA), as previously described [33].

#### 2.10.1. Alkaline Comet Assay

Induction of DNA strand breaks (SBs) and alkali labile sites (ALS) on 16HBE14o− cells was assessed by the alkaline comet assay (pH > 13), as previously described [34]. 16HBE14o− cells were seeded in 48-well plates at the density of 1.5 × 10^5^ cells/well and incubated for 24 h at 37 °C and 5% CO_2_. Then, cells were exposed to GBMs (1.5–50 µg/mL) for 3 and 24 h. As a positive control, 20 mM H_2_O_2_ (Riedel–de Haen; Seelze, Germany) was used. After treatment, cells were washed three times with warm MEM (Minimum Essential Medium; Gibco, Paisley, UK) containing 50% FBS (Gibco, Paisley, UK) under agitation (75 rpm, 5 min). After washing, cells were detached with 1% trypsin-EDTA (Biowest; Nuaillé, France), centrifuged (5 min, 1.1 rfc) and the supernatant was discarded. Cell pellet was mixed with 0.7% low melting point agarose (Merck; Darmstadt, Germany) and two drops of 25 µL of the cell suspension was placed on top of each pre-coated slide (1% standard agarose; VWR, Radnor, PA, USA). A 20 × 20 mm coverslip was placed on top of each drop. After removing the coverslips, slides were kept immersed for 30 min at 4 °C in a lysis solution composed of 2.5 M NaCl (Merck; Darmstadt, Germany); 0.1 M EDTA (Merck; Darmstadt, Germany); 10 mM Tris (Roche; Basel, Switzerland); 1% Triton X-100 (Thermo Fisher Scientific; Hampton, NH, USA); and adjusted to pH 10 with NaOH (Merck; Darmstadt, Germany). After the lysis process, the slides were subjected to an electrophoresis (1 V/cm, 15 min, 4 °C) and neutralized by washing them with PBS (Gibco; Paisley, UK), followed by distilled water (10 min, 4 °C each wash). Finally, the slides were coded, stained with ethidium bromide (VWR; Radnor, PA, USA) to analyze the comets with a fluorescence microscope (Axioplan 2; Zeiss, Jena, Germany) and a comet counter (Komet 5.5; Kinetic Imaging Ltd., Liverpool, UK). 

A total of 50 cells per slide, two slides per replicate and two replicates per dose were scored (200 cells per dose and experiment were analyzed). Data were expressed as the mean ± SD of two independent experiments performed in duplicate; the induction of SBs and ALS are reported as % of DNA in tail.

#### 2.10.2. Cytokinesis-Block Micronucleus Test 

Induction of micronucleus (MN) by GBMs on 16HBE14o− cells was assessed by the Cytokinesis-Block Micronucleus (CBMN) test, following the principles of the OECD TG 487 [32] and as previously described by Lindberg and colleagues [35]. The 16HBE14o− cells were seeded in 6-well plates at a density of 5 × 10^5^ cells/well. After 24 h of incubation at 37 °C and 5% CO_2_, the cells were exposed to GBMs (1.5–50 µg/mL) for 24 h. Mitomycin C (Sigma–Aldrich Chemie; Steinheim, Germany) at 50 ng/mL was used as a positive control. After treatment, the cells were washed three times (MEM with 50% FBS, 37 °C, 5 min, 700 rpm) and incubated with 6 µg/mL cytochalasin B (Sigma–Aldrich Chemie; Steinheim, Germany) for ~1.5 cell cycles (40 h). Then, cells were washed with PBS, detached with 1% trypsin-EDTA and collected. After centrifugation (110 rpm, 5 min), 5 mL of hypotonic solution (50% RPMI; Gibco, Paisley, UK; 50% H_2_O) was added fast and vigorously to each tube that was incubated for 2 min. The cells were then centrifugated, fixed in methanol and acetic acid (Merck; Darmstadt, Germany) 3:1 and subsequently in 97% methanol and 3% acetic acid. One or two drops of cell suspension were spread on a frosted slide (VWR, Radnor, PA, USA). Three slides were prepared per replicate and allowed to dry at room temperature. The slides were stained by immersion in acridine orange (0.003%; Sigma–Aldrich Chemie; Steinheim, Germany) and subsequently washed three times in cold Sörensen buffer (4.61 mg/mL KH_2_PO_4_, 5.84 mg/mL Na_2_HPO_4_ × 2H_2_O; Merk, Darmstadt, Germany) at 4 °C for 3 min. The slides were then stained with 4′6-diamidino-2-phenylindole (DAPI, 1 mg/mL; Thermo Fisher Scientific, Hampton, NH, USA) for 5 min in the dark and rinsed under tap water. The slides were analyzed with a fluorescence microscope (Axioplan 2; Zeiss, Jena, Germany). 

MN induction data were expressed as the mean ± SD of one independent experiment performed in duplicate, reported as an MN fold increase compared to the negative control. The frequency of micronucleated cells was scored in a total of 2000 binucleated cells/replicate and 4000 cells/dose.

As requested by the OECD TG 487, cytotoxicity was assessed in the same slides used for the MN analyses, to ensure that the doses applied did not exceed 55 ± 5% cytostasis [32,36]. The replication index (RI), which is one of the recommended measures, was determined through the cytokinesis-block proliferation index (CBPI) from 200 cells per dose (100 cells/replicate) as follows:

CBPI = (M1 + 2 × B + 3 × (T + Q + Mu))/200(1)

% Cytostasis = 100 − 100 × [(CBPI_treated_ − 1)/(CBPI_control_ − 1)] (2)

RI = 100 − % Cytostasis(3)

where M, B, T, Q and MU represent the number of cells that are mono, bi, tri, quad and multi nucleated, respectively.

### 2.11. Statistical Analysis

Non-linear regression of concentration-effect data was performed using the GraphPad Prism version 8.0.2, which was used also to calculate the concentration giving 50% of the effect (EC_50_), while the heatmap and clustering analysis were performed using the software RStudio PBC. 

Regarding TEM analysis, statistical differences between average lateral dimensions of the materials were calculated using the Student’s *t*-test. For all the other data, the statistical analysis was performed by the GraphPad Prism software (PrismGraphPad, Inc.; Boston, MA, USA) and significant differences were considered at *p* < 0.05. In particular: (i) cytotoxicity data obtained by comparative evaluation of different GBMs were analyzed by a two-way analysis of variance (ANOVA), followed by Bonferroni’s post-test (GraphPad Prism, V.8.0.2); (ii) data obtained by cytokines quantification were analyzed by a one-way ANOVA analysis, followed by Bonferroni’s post-test (GraphPad Prism, V.8.0.2); (iii) data obtained by the comet assay and the CBMN test were analyzed by one-way ANOVA, followed by Dunnett’s multiple comparisons test (GraphPad Prism, V.9.3.1), in comparison to the corresponding negative control, and by linear regression analysis; the positive controls were evaluated in comparison to their corresponding negative control by the unpaired one-tailed *t*-test.

## 3. Results

### 3.1. Physicochemical Characterization of GO, rGO and wdGO

The physicochemical properties of each material were characterized by elemental analysis, TGA, XRD and TEM. The elemental analysis data are presented in Table 1. The oxidation level of GO and wdGO was clearly higher than that of rGO. It can also be appreciated that the graphene oxides contain a low percentage of sulfur, which came from the synthesis process. During the reduction process, this sulfur was removed.

**Figure 1:**
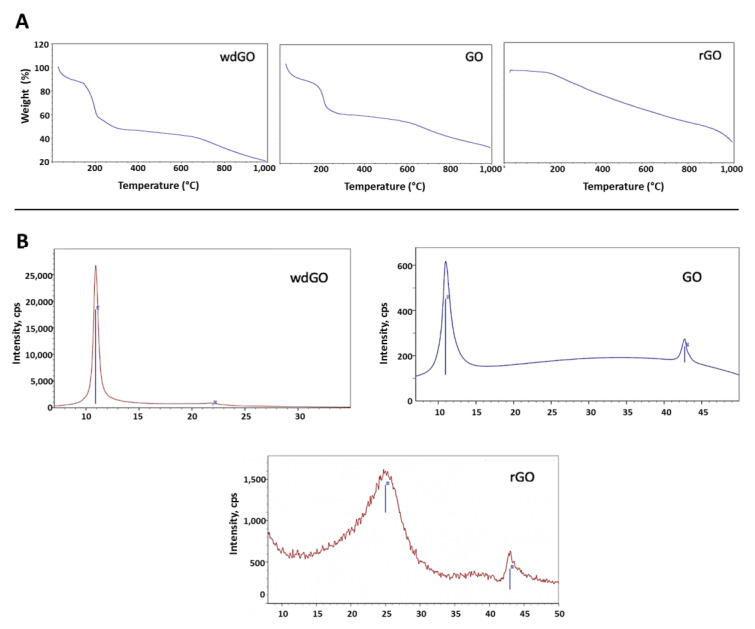
Physicochemical characterization of GO, wdGO and rGO. (**A**) TGA spectra; (**B**) XRD spectra.

TGA spectra (Figure 1A) of the two graphene oxides was characterized by weight loss from 0 to 125 °C, corresponding to the adsorbed water, and a second loss from 125 °C to 300 °C, which includes decomposition of the organic moieties (GO and wdGO). In the case of rGO, its thermal stability was much higher, and the decomposition of the oxygenated functionalities, which are still present, occurred in a more gradual manner. 

XRD spectra of each material is presented in Figure 1B. The graphite (002) peak at 26° disappeared, reflecting the total conversion of the reaction. The GO (001) peak at 11° was translated in an interplanar distance of 8Å. When reducing GO, the interlayer distance decreases, and the peak was moved to higher degrees. The crystallinity also was lower due to the defects that were formed in the carbonous structure. The most crystalline material was wdGO, which was dried for the characterization. For wdGO, the FWHM of the peak is smaller than that of the GO in powder format, an indicator of its higher crystallinity. On the contrary, rGO is the less crystalline material presenting a very broad peak, around 22°, due to the loss of part of the functional groups that decreases the interlayer distance.

**Figure 2:**
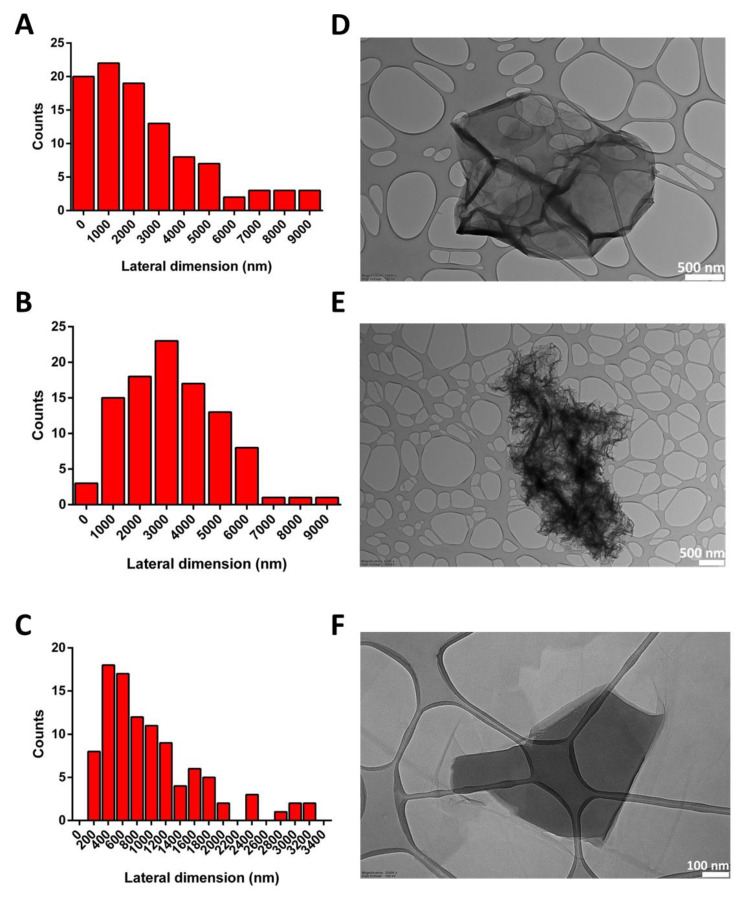
Lateral dimension distribution calculated from TEM images (**A**–**C**) and representative TEM images (**D**–**F**) of GO (**A**,**D**), rGO (**B**,**E**) and wdGO (**C**,**F**). Scale bar: 500 nm (**D**,**E**); 100 nm (**F**).

The lateral dimensions of all materials were computed by TEM analysis, after the examination of 100 different sheets for each material. The lateral size distribution was located between 124 and 8792 nm, with an average lateral dimension of 2476 ± 2266 nm for GO (Figure 2A); between 376 and 8552 nm, with an average lateral dimension of 3231 ± 1763 nm for rGO (Figure 2B); and between 127 and 3256 nm, with an average lateral dimension of 1005 ± 709 nm for wdGO (Figure 2C). Despite the different average lateral dimensions of the three materials, these did not appear significant (*p* > 0.05; Student’s *t*-test), given their wide lateral size distributions. Representative TEM images of materials are shown in Figure 2. 

The stability of each material dispersion (1 mg/mL) was assessed by UV-visible spectrophotometric analysis, measuring absorbance at 660 nm up to 24 h. Regarding wdGO, flakes dispersion appeared almost completely stable up to 24 h. Contrarily, with regard to GO and rGO dispersions, their stability was almost maintained within the first two hours, but heavily reduced after 12 and 24 h (Supplementary Materials, Figure S1).

**Figure 3:**
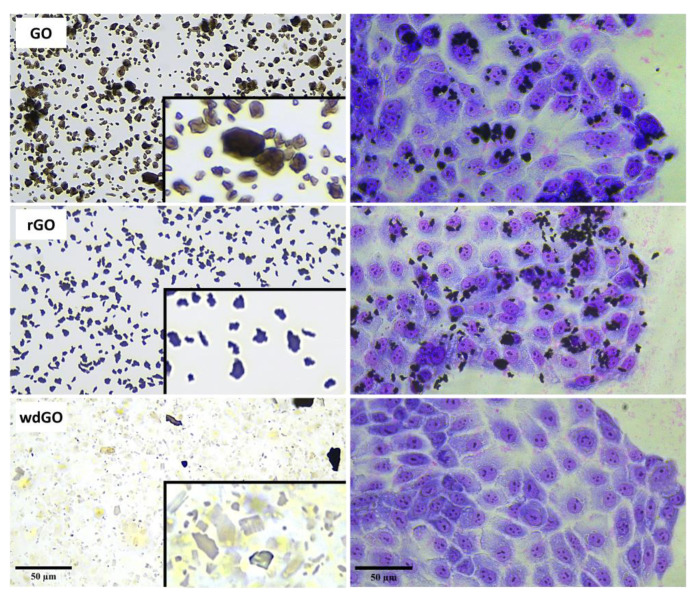
Representative images of GO, rGO and wdGO dispersed in cell culture media (10 µg/mL) and representative images of May–Grunwald/Giemsa-stained 16HBE14o− cells exposed to each material (10 µg/mL) for 24 h. Images were captured with an inverted optical microscope at a 40× magnification. Scale bar: 50 µm. In the boxes a further zoom of each image of GO, rGO and wdGO dispersed in cell culture media is reported.

Material shape and geometry (10 µg/mL) were analyzed by light microscopy in a cell culture medium (Figure 3). Powdered GO was characterized by smooth edges and regular structures. However, rGO showed an irregular morphology characterized by sharp, rough and irregular edges. Lastly, wdGO showed a heterogeneous geometry, with some flakes appearing sharp-edged, but with relatively regular structures as compared to rGO, while others were more similar to smooth-edged GO flakes. Images of 16HBE14o− cells exposed to each material (10 µg/mL) for 24 h are also shown in Figure 3. Images suggest a stable interaction between cells and GO or rGO, not excluding the possibility that some GO or rGO flakes might be internalized inside cells. Contrarily, in case of wdGO, cytochemical analysis detected only weak signals ascribable to the material, which was also due to the higher optical transparency of the flakes as compared to GO and rGO. The analysis suggests very weak interaction between wdGO and cells, as compared to the other materials. In addition, no obvious morphological alterations can be observed in May–Grunwald/Giemsa-stained cells after exposure to 10 µg/mL GBMs.

### 3.2. Cellular Internalization of GO, rGO and wdGO

**Figure 4:**
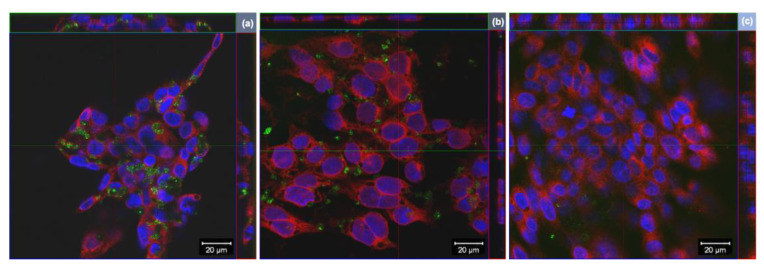
Representative images of orthogonal view of confocal images of 16HBE14o− cells exposed to 25 µg/mL of (**a**) GO, (**b**) rGO or (**c**) wdGO for 24 h. Cell nuclei: blue; cell membranes: red; graphene materials: green. Images were captured with a confocal laser scanning microscope at a 40× magnification. Scale bar: 20 µm.

Cells internalization of GO derivatives was further studied by laser confocal microscopy, exploiting light reflection properties of GBMs. Representative images, reconstructed offline by merging red fluorescence (plasma membranes), blue fluorescence (nuclei) and green signal (light reflected by GO derivatives), are shown in Figure 4. Images confirm the internalization of GO and rGO into bronchial cells, whereas there does not appear to be significant uptake of wdGO. A similar pattern of cell interaction could be observed for GO and rGO, with most of the signals observed in the cytoplasm with varying degrees of patchiness.

### 3.3. Detection of Endotoxin Contamination

To exclude any endotoxin contamination of GBMs, a TET assay was performed on macrophages obtained by differentiation of THP-1 cells. Macrophages were exposed to a non-cytotoxic concentration of each GBM (1 µg/mL) for 24 h, in the presence or absence of polymyxin B sulfate (10 µM); LPS (100 ng/mL) was included as a positive control. Afterward, cell media were collected and TNF-α was quantified by a specific ELISA; a standard curve generated on the basis of LPS-induced TNF-α release by macrophages was used for the quantitation of LPS present in each GBM.

**Figure 5:**
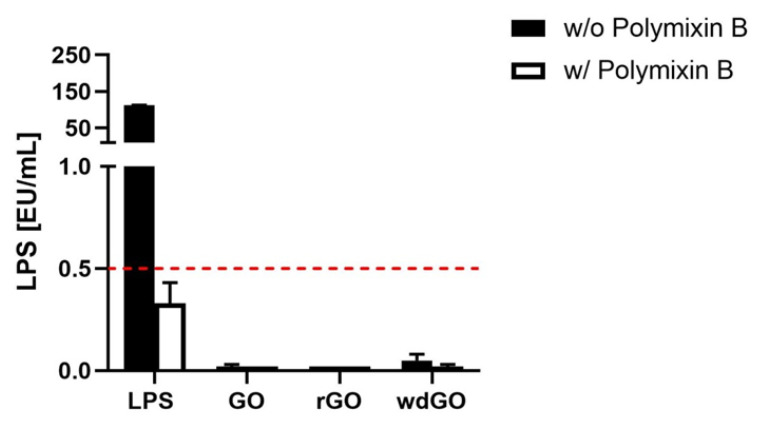
Endotoxin detection in GBMs using the TNF-α expression test (TET assay). Macrophages obtained by differentiation of the human monocytic THP-1 cell line were exposed for 24 h to GO, rGO or wdGO at a non-cytotoxic concentration (1 µg/mL) in the presence or absence of polymyxin B sulfate (10 µM). LPS (100 ng/mL) was included as a positive control. The red dashed line represents the 0.5 EU/mL acceptable limit suggested by the U.S. FDA. Results are the mean ± SE of three independent experiments.

If LPS quantitation in macrophages exposed to GBM is equivalent in presence or absence of polymyxin B sulfate, then the material is not endotoxin contaminated, and the observed effect is an intrinsic feature of the material. On the contrary, the material is endotoxin contaminated if LPS quantitation in presence of polymyxin B sulfate is significantly reduced or even abolished. The results, expressed as endotoxin units/mL (EU/mL), are shown in Figure 5. According to U.S. Food and Drug Administration (FDA) guidelines, 0.5 EU/mL is the endotoxin limit for considering a material not contaminated by endotoxin in regards of medical devices [37]. None of the tested materials showed levels of LPS above 0.5 EU/mL; in addition, LPS quantified in presence of polymyxin B sulfate was similar to that quantified in its absence, confirming that no GBM was endotoxin contaminated.

### 3.4. Effects on Cell Viability

The effect of GO, rGO and wdGO (0.4–100.0 μg/mL) on 16HBE14o− cells viability was assessed after 3 and 24 h exposure by the WST-8 assay. Data are reported as percentage of cell viability in cells exposed to GBMs with respect to untreated control cells (100% cell viability). The effect of GO was compared to that of rGO and wdGO to evaluate the impact of chemical reduction and formulation on its cytotoxic potential, respectively.

**Figure 6:**
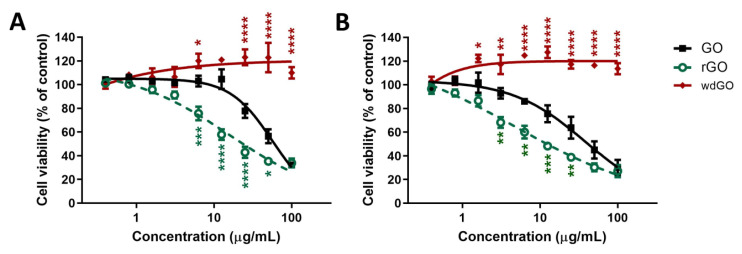
Effects of GO, rGO and wdGO on 16HBE14o− cells viability evaluated by the WST-8 assay after 3 h (**A**) and 24 h (**B**) exposure. Data are reported as % of cell viability in cells exposed to GBMs with respect to untreated control cells (negative control) and are the mean ± SE of three independent experiments performed in triplicate. Statistical differences vs. GO: * *p* < 0.05; ** *p* < 0.01; *** *p* < 0.001; **** *p* < 0.0001 (two-way ANOVA and Bonferroni’s post-test).

Figure 6 shows the effects of GO, rGO and wdGO on cell viability after a short (3 h; panel A) and a longer (24 h; panel B) exposure. After 3 h exposure, GO reduced cell viability starting from the concentration of 25 μg/mL (78% cell viability with respect to untreated controls), with an EC_50_ value equal to 56.4 μg/mL (95% confidence intervals, CI = 47.8–66.6 μg/mL). However, rGO was cytotoxic even at 6.3 μg/mL (76% cell viability), exerting a cytotoxic effect significantly higher than that of GO (*p* < 0.001); its EC_50_ value was equal to 16.23 μg/mL (95% CI = 9.5–27.6 μg/mL), 3.5-fold lower than that of GO. wdGO did not reduce cell viability, but rather slightly increased it, starting from the concentration of 6.3 μg/mL (120% cell viability). The increased cell viability by wdGO was significant as compared to GO effect (*p* < 0.05), and its maximum effect (123% cell viability) was exerted at a concentration of 50 μg/mL (*p* < 0.0001).

After 24 h exposure, a similar trend was observed. GO reduced cell viability at the concentration of 6.3 μg/mL (86% cell viability) and above, with an EC_50_ value of 38.3 μg/mL (95% CI = 27.0–54.4 μg/mL). The effect of rGO was significantly higher, starting at the concentration of 3.1 μg/mL (68% cell viability; *p* < 0.01), with an EC_50_ value of 4.8 μg/mL (95% CI = 1.1–20.6 μg/mL), almost eight-fold lower than that of GO. By contrast, also in this case, wdGO did not reduce cell viability, but induced a slight increase at a concentration of 1.6 μg/mL (122% cell viability; *p* < 0.05) and above.

OVerall, these results suggest the following rank of cytotoxic potency: rGO > GO >> wdGO.

### 3.5. ROS Generation

The effects of GO, rGO or wdGO (0.4–100.0 μg/mL, up to 24 h exposure) on ROS production were evaluated by the DCFDA assay through a comparative approach. An initial set of experiments was carried out to evaluate the kinetics of ROS production in 16HBE14o− cells exposed to each material for increasing times (45 min up to 24 h exposure); cells exposed to 1 mM AAPH were used as positive control. Each material induced a concentration- and time-dependent ROS production, but significantly lower compared with the positive control starting from 6 h exposure. In general, comparing the whole kinetic profiles, rGO was the most potent material in inducing ROS production after 1.5 h exposure, whereas GO showed a potency comparable to that of wdGO (Supplementary Figure S2).

**Figure 7:**
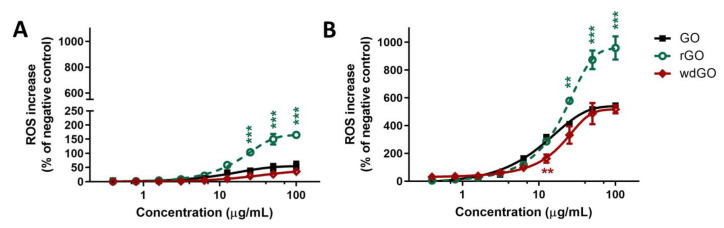
ROS production in 16HBE14o− cells after exposure to GO, rGO or wdGO for 3 h (**A**) or 24 h (**B**), evaluated by the DCFDA assay. Results are expressed as percentage of ROS increase with respect to negative control (cells not exposed to GBMs) and are the mean ± SE of at least three independent experiments performed in triplicate. Statistical differences vs. GO: ** *p* < 0.01; *** *p* < 0.001 (two-way ANOVA and Bonferroni’s post-test).

To better assess the potencies of the three materials as ROS production inducers, the effects of 3 h and 24 h exposure to rGO or wdGO were compared to that of GO, as shown in Figure 7. Data are reported as percentage of ROS increase with respect to negative control (cells not exposed to GBMs). As reported in panel (A), 3 h exposure to rGO induced a ROS production significantly higher than that induced by GO, starting from the concentration of 25 μg/mL (104% and 39% increase of ROS production, respectively; *p* < 0.001) up to 100 μg/mL (165% and 56% increase of ROS production, respectively; *p* < 0.001). As compared to GO, wdGO induced a slightly lower effect, but the difference was not significant (36% increase of ROS production at the highest concentration, 100 μg/mL). A similar trend was observed also after 24 h exposure (panel B): as compared to GO, rGO induced a significant higher ROS production starting from the concentration of 25 μg/mL (410% and 578% of ROS production, respectively; *p* < 0.01) up to 100 μg/mL (544% and 958% of ROS production, respectively; *p* < 0.001). wdGO induced ROS production with a slightly lower potency than GO, the difference being significant only at the concentration of 12.5 μg/mL (162% and 318% increase of ROS production, respectively; *p* < 0.01). Thus, these results suggest that rGO is the most potent material in inducing ROS production, followed by GO and wdGO, with the latter showing a barely lower effect with respect to GO. 

### 3.6. Pro-Inflammatory Mediators Release

To assess the pro-inflammatory effects of each material in 16HBE14o− cells, a panel of inflammatory mediators (IL-1α, IL-1β, IL-6, IL-8, IL-18, TNF-α, GM-CSF, INF-γ, MCP-1, RANTES, ECF/CCL11, PG-E_2_) was quantified in cell media collected after 24 h cells exposure to GO, rGO or wdGO (10 μg/mL), using specific ELISA assays. 

**Figure 8:**
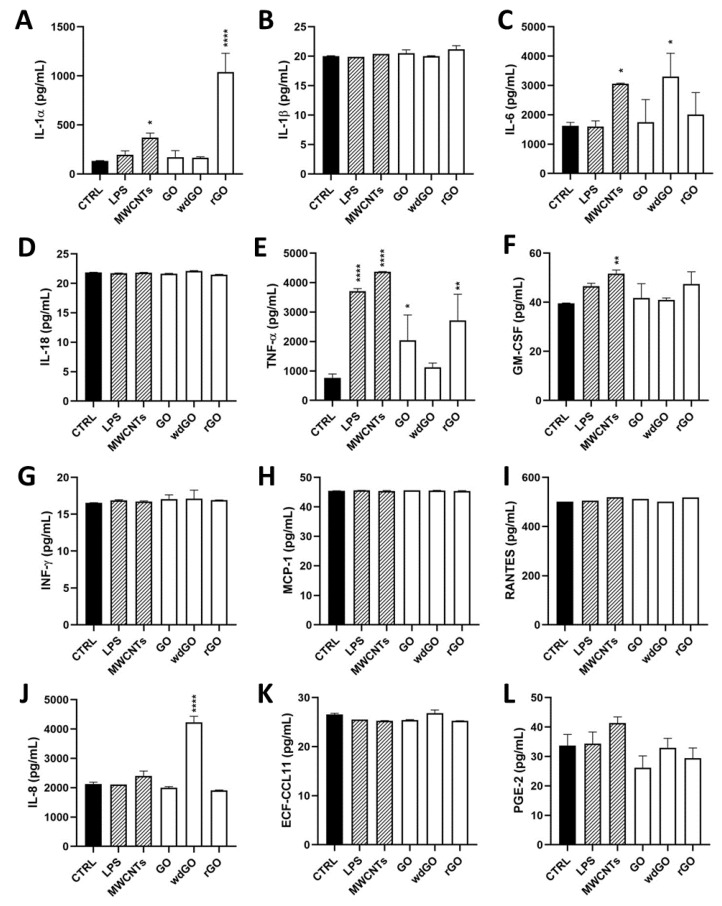
Profile of pro-inflammatory mediators released by 16HBE14o− cells exposed to GO, rGO or wdGO (10 μg/mL) for 24 h. After 24 h exposure, supernatants were collected and IL-1α (**A**), IL-1β (**B**), IL-6 (**C**), IL-18 (**D**), TNF-α (**E**), GM-CSF (**F**), INF-γ (**G**), MCP-1 (**H**), RANTES (**I**), IL-8 (**J**), ECF/CCL11 (**K**), PG-E_2_ (**L**) were measured by specific ELISA assays. Cells were exposed to LPS 1 µg/mL as a positive control or MWCNT Mitsui-7 1 µg/mL as a reference material. The data, reported as pg/mL of cytokines released in the media, are the mean ± SE of three independent experiments. Statistical differences vs. negative controls: *, *p* < 0.05; **, *p* < 0.01; ****, *p* < 0.0001 (one-way ANOVA and Bonferroni’s post-test).

Figure 8 shows the amount of the specific mediators, expressed as pg/mL, released by 16HBE14o− cells after GBM treatment, in comparison with untreated cells (negative control), the positive control (cells exposed to 1 μg/mL LPS) and the reference material (cells exposed to 1 μg/mL MWCNT Mitsui-7). As compared to the negative control, significant differences were observed only for IL-1α, IL-6, IL-8 and TNF-α. Considering IL-1α, as compared to the negative control (134 pg/mL), only rGO significantly increased its release to 1038 pg/mL (7.7-fold increase; *p* < 0.0001), at a level higher than that induced by LPS (197 pg/mL; 1.5-fold increase) or MWCNT Mitsui-7 (371 pg/mL; 2.8-fold increase). Regarding TNF-α, rGO significantly increased its release (2721 pg/mL; 3.5-fold increase; *p* < 0.01) with respect to negative controls (770 pg/mL), even if was at a level lower than that induced by LPS (3715 pg/mL; 4.8-fold increase) or MWCNT Mitsui-7 (4368 pg/mL; 5.7-fold increase). Similarly, even if it was at a level lower than that induced by rGO, GO significantly increased TNF-α release (2045 pg/mL; 2.6-fold increase; *p* < 0.05) with respect to negative controls. Regarding IL-6, as compared to the negative control (1624 pg/mL), only wdGO significantly increased its release to 3308 pg/mL (2-fold increase; *p* < 0.05)—an effect similar to that induced by the reference MWCNT Mitsui-7 control (3057 pg/mL; 1.9 folds increase). Similarly, wdGO was the only material able to significantly increase IL-8 release (4232 pg/mL; 2-fold increase; *p* < 0.0001) with respect to negative controls (2129 pg/mL). 

**Figure 9:**
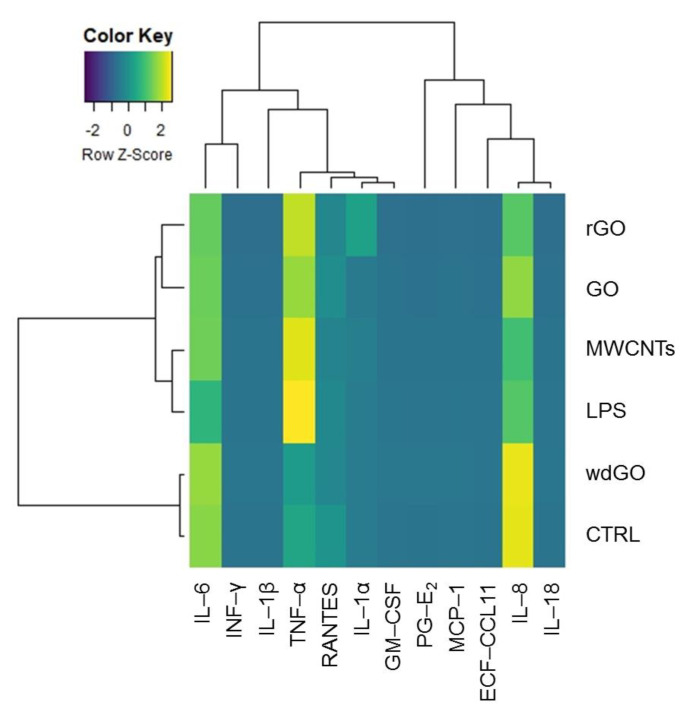
Heatmap representing the release of pro-inflammatory mediators by 16HBE14o− cells exposed to GO, rGO or wdGO (10 μg/mL) for 24 h, with related clustering analysis. Cells were exposed to LPS (1 µg/mL) as positive control or to MWCNT Mitsui-7 (1 µg/mL) as reference material. Colors indicate the release levels of each mediator (yellow indicates a major release, blue indicates a minor one). The dendrograms represent the similarity between the different samples analyzed: the branch lengths are proportional to the similarities between samples, the shorter branch indicating closer relationships.

To identify any similarity between the pattern of pro-inflammatory release induced by GBM treatment with negative control, positive control or the reference material, the amount of each mediator (pg/mL) released in culture media by untreated cells, cells exposed to GO, rGO or wdGO, and those treated with the positive control (LPS) or the reference material (MWCNT Mitsui-7), were displayed on a heatmap (Figure 9). A clustering analysis was performed, in which dendrograms represent the similarity between the different samples analyzed: the branch lengths are proportional to the similarities between samples, with the shorter branch indicating closer relationships. Three major clusters were identified: (i) the first one, including the negative control and cells exposed to wdGO with a high similarity degree, suggesting a cytokines release pattern for the latter comparable to that of untreated cells; (ii) the second one, including rGO and GO samples; and (iii) a third one, including the positive control (LPS) and the reference material (MWCNT Mitsui-7). Data of cells treated with GO or rGO segregated with the last cluster—even if they had a very low affinity degree, but not with those of untreated controls—suggested a pattern of pro-inflammatory release barely similar to that of the positive control and the reference material, rather than that of the negative control. Hence, in line with the previous results, the clustering analysis suggests that wdGO is the least inflammogenic material compared to GO and rGO.

### 3.7. Genotoxicity

The concentrations of GBMs tested for their genotoxic potential were selected on the basis of a preliminary cytotoxicity assay, as recommended by the OECD TG 487 [32]. According to the results summarized in Supplementary Figure S3, the limit of 45 ± 5% cell viability after 24 h exposure (corresponding to the 55 ± 5% cytotoxicity established in OECD test guidelines) corresponded to a concentration of 50 µg/mL of rGO, which was the most cytotoxic material. Hence, this concentration was chosen as the highest tested dose for all the GBMs.

#### 3.7.1. Alkaline Comet Assay 

**Figure 10:**
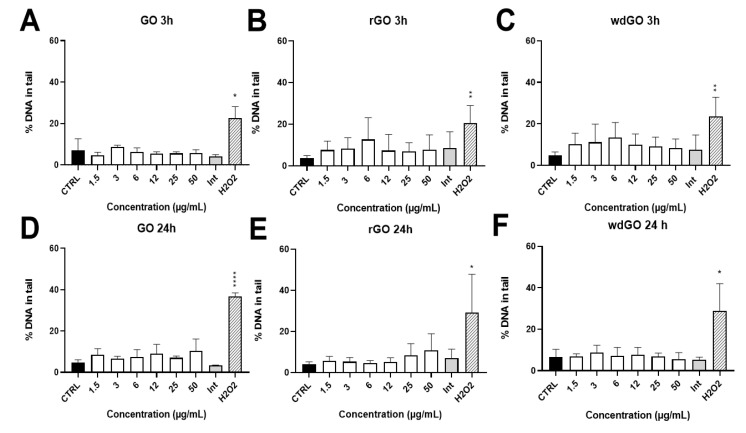
Induction of DNA damage in 16HBE14o− cells evaluated by the alkaline comet assay after treatment with GO at 3 h (**A**) and 24 h (**D**), rGO at 3 h (**B**) and 24 h (**E**) and wdGO at 3 h (**C**) and 24 h (**F**). Results are expressed as percentage of DNA in tail. The mean ± SD of two independent experiments is represented. CTRL: negative control; Int: interference at 50 μg/mL. Statistical differences of 1.5–50 µg/mL and Int vs. CTRL (one-way ANOVA and Dunnett’s multiple comparisons test); H_2_O_2_ vs. CTRL (unpaired one-tailed *t*-test): *: *p* < 0.05; **: *p* < 0.01; ****: *p* < 0.001. A significant linear dose-response (*p* < 0.005, slope = 0.129) was observed after 24 h treatment with rGO (**E**).

The induction of SBs and ALS in 16HBE14o− cells exposed to GO, rGO or wdGO (1.5–50.0 μg/mL) for 3 h and 24 h was assessed by alkaline comet assay. The results are shown in Figure 10. None of the materials showed interferences with the assay up to 50 μg/mL (interference control). No significant increase of DNA damage over the negative control value was observed for any of the materials and concentrations tested. After 24 h exposure, while rGO showed a slight, but significant (*p* < 0.005, slope = 0.129), linear concentration-response effect, the other treatments showed no linear concentration responses.

#### 3.7.2. Cytokinesis-Block Micronucleus Test

**Figure 11:**
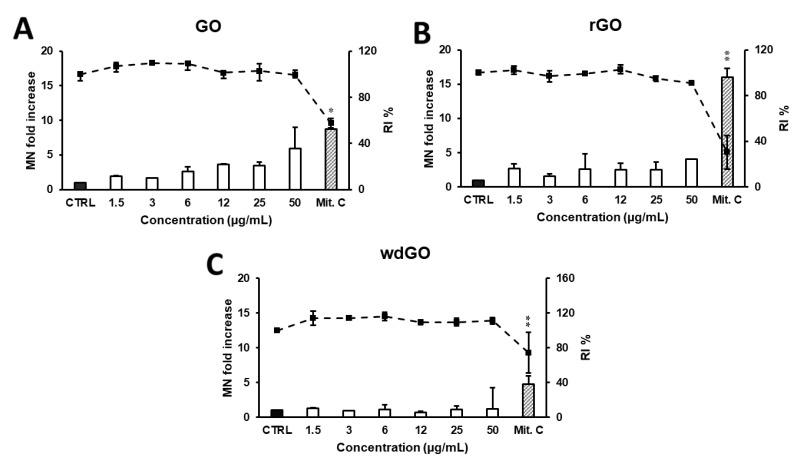
Induction of MN in 16HBE14o− cells evaluated by the CBMN test after 24 h treatment with GO (**A**), rGO (**B**) or wdGO (**C**). Results are expressed as micronuclei (MN) fold increase (bars) and replication index (RI) % (line). The mean ± SD of one experiment carried out in duplicate is represented. CTRL: negative control; Mit. C: mitomycin C. Statistical differences of 1.5–50 µg/mL vs. CTRL (one-way ANOVA and Dunnett’s multiple comparisons test); Mit.C vs. CTRL (unpaired one-tailed *t*-test): *, *p* < 0.05; **, *p* < 0.01. A significant linear dose-response (*p* < 0.005, slope = 0.086) was observed for GO (**A**).

The induction of MN in 16HBE14o− cells exposed to GO, rGO or wdGO (1.5–50.0 μg/mL) for 24 h was evaluated by CBMN test. As shown in Figure 11, a reduction in RI below 45 ± 5% of the concurrent negative control (corresponding to 55 ± 5% of cytotoxicity, as set by the OECD guidelines) was induced by none of the GBMs, at none of the tested concentrations. None of the materials induced a significant increase in the frequency of MN compared with the negative control at any of the tested doses, although a significant linear concentration response (*p* < 0.005, slope = 0.086) was found for GO.

## 4. Discussion

In recent years, GBMs have attracted considerable attention for their promising applications in various disciplines, from nanotechnology to biomedicine [2,11]. In particular, the literature data suggest that GBM physicochemical properties may influence their biocompatibility and biological effects, including their toxic potential [12,38,39].

Hence, this study focused on the impact of chemical reduction and formulation of GO on its toxic potential. To this end, we evaluated the effects of GO prepared as a powder or in a stable water dispersion form on 16HBE14o− bronchial epithelial cells to study the impact of formulation on the cytotoxicity, and the effects of its chemically reduced form (rGO) to investigate the influence of chemical reduction. All these materials were tested by the TET assay that excluded any endotoxin contamination, which may be a confounding factor in the assessment of the cytotoxic effects of such materials [40,41]. We have also used materials characterized by a similar lateral dimension profile, to exclude any additional bias due to different sizes—an important property that may affect the cytotoxic potential of GO [15]. Indeed, even though differences in the average lateral dimensions of the three materials were noticed, these did not appear significant due to their wide lateral size distributions, as expected for these materials. Then, GBM cytotoxic potential on bronchial cells was investigated after a short (3 h) and a longer (24 h) exposure time by means of cell viability reduction (WST-8 reduction assay), ROS generation (DCFDA assay), release of pro-inflammatory mediators as well as DNA and chromosome damage (Alkaline Comet assay and Cytokinesis-Block Micronucleus test). 

GO induced a concentration-dependent reduction of cell viability, with an EC_50_ value of 56.4 μg/mL and 38.3 μg/mL after 3 and 24 h exposure, respectively. A previous study of A549 cells suggests GO as a highly biocompatible material, with no obvious cytotoxicity. At low concentrations, no cell viability reduction was observed, whereas increasing the concentration up to 200 μg/mL, GO induced a slight cell viability reduction [42]. GO cytotoxicity was previously also evaluated using primary, non-transformed normal human bronchial epithelial (NHBE) cells. Exposure of NHBE cells to GO (0.05–100 μg/mL) for 24 h or 7 days induced a significant cell viability reduction only after 7-day exposure to the concentration of 0.5 μg/mL and above, provoking up to 90% cell viability reduction at the highest concentrations (50–100 μg/mL). After 24 h exposure, GO induced only a slight, but not significant, reduction of cell viability, in disagreement with our results [43]. Considering that oxidative stress is proposed as a key mechanism involved in the toxicity of various nanomaterials [44,45,46], in the present study, GBMs were evaluated for their ability to increase ROS levels in 16HBE14o− cells up to 24 h exposure. GO increased ROS production in a concentration- and time-dependent manner, but its effect was significantly lower than that induced by the positive control, suggesting a moderate oxidative potential. These results are in line with previous findings on A549 and BEAS-2B cells exposed to GO at concentrations and exposure times comparable to those used in our study [25]. ROS overproduction in A549 cells exposed to GO for 24 h was already reported by Chang and co-workers, who showed a concentration- and GO size-dependent effect. Interestingly, despite the ROS level in GO-treated cells (200 µg/mL) being 2.1 to 3.9 times higher than that measured in negative control cells, depending on the material size, the ROS level for the positive control was 12.0 times higher than that of the negative control. This level was much higher than that recorded in GO-treated cells, under the same conditions, in agreement with our results [42].

To investigate the influence of GO chemical reduction on its toxic potential, we evaluated the effects of its chemically reduced form rGO. Also, rGO induced a concentration-dependent reduction of cell viability, with a potency significantly higher than that of GO: after 3 and 24 h exposure, it induced a cytotoxic effect with EC_50_ values 3.5-fold and 8-fold lower than those of GO, respectively. The concentration-dependent cell viability reduction by rGO is in agreement with a previous in vitro study on A549 cells, in which 50 and 250 μg/mL rGO reduced living cells to 50% and 40%, respectively [47]. The cytotoxic potential was confirmed by the increased ROS production, with rGO inducing a significantly higher effect than GO. Mittal and co-workers previously assessed the effects of GO and two different rGO, a thermally reduced GO (TRGO) and a chemically reduced GO (CRGO) in BEAS-2B and A549 cells, through a comparative approach. Despite all the materials inducing an oxidative stress-mediated cytotoxicity in both cell lines, the effect of TRGO was higher than that of GO and CRGO, both in terms of ROS production and cytotoxicity. The higher effect was probably due to the small lateral dimension and sharp corners of TRGO, favoring its cellular internalization [25]. In this case, significant differences between rGO obtained by the two diverse reduction procedures can be identified, suggesting a key role of the selected reduction process in the toxic effects. Considering the materials tested in our study, the higher cytotoxic potential of rGO, as compared to that of GO, could be related to an enhanced physical–mechanical injury at the cellular level, consequent to rGO interaction with cell membrane. Indeed, as demonstrated by our results, rGO presents a wrinkled and twisted structure with sharp edges, whereas GO is characterized by smoother and rounded edges. The physical interactions of GBMs with cell membranes are one of the main causes of GBM cytotoxicity and ROS production, which could also be generated by the material’s reaction with a cell surface, leading to lipid peroxidation [48]. rGO appears to possess high affinity to cell membranes and, due to its irregular and sharp edges, it can affect their integrity. Moreover, strong conditions usually used in chemical methods employed in rGO production can influence its structure and its biological activity [49]. In our study, we tested an rGO obtained by the chemical reduction of GO using ascorbic acid. The cytotoxic effects of ascorbic acid-reduced GO (AA.rGO) have been studied in a previous study in comparison to those of GO and hydrazine-reduced GO (H.rGO), hydrazine being a well-established reagent for rGO generation. The study highlighted diverse cytotoxic effects on A549 lung cells, identifying AA.rGO as the least biocompatible material, and suggested physical membrane damage as the primary mechanism of cytotoxicity. Indeed, AA.rGO was characterized by an irregular and wrinkled shape, resulting from the use of ascorbic acid as a reducing agent, in line with the findings of our study. The interaction of AA.rGO sharp edges with A549 cells may impair cell membrane integrity via physical disruption, leading to a loss of cell stability and ultimately to cell death [50].

Lastly, to investigate the impact of GO formulation on its cytotoxic potential, we compared the effects of GO prepared as a powder (GO) to that of GO in a stable water dispersion form (wdGO). In contrast to GO, both 3 and 24 h 16HBE14o− cells exposure to wdGO did not reduce cell viability, but rather slightly increased it, suggesting an extremely weak cytotoxic potential. In addition, wdGO induced ROS production in a time- and concentration-dependent way, but its effect was slightly lower than that of GO, after 24 h exposure. The lower cytotoxic potency of wdGO, especially in terms of lack of cell viability reduction, could be probably due to the stability of its dispersion, significantly higher than that of GO and rGO dispersions, and almost complete up to 24 h. Being the material in a stable dispersion, its deposition above cells and its subsequent interaction with cell membranes, appeared heavily limited, as suggested by the images acquired by optical microscope. Indeed, given the number and intensity of the repeated washings before acquisition of images, our cytochemical analysis certainly demonstrates that wdGO cellular interaction is extremely weak or even absent with respect to GO and rGO. In addition, confocal microscopy analysis demonstrated that wdGO was the only material not interacting with cells nor being internalized. Hence, the consequent cell membranes physical disruption could be hampered, possibly explaining the lower cytotoxic effects in comparison to GO and rGO.

Given the ability of all the materials to increase ROS production in bronchial cells, in the second part of the study, we evaluated their effects by means of inflammatory response and genotoxicity, given their well-known correlation with oxidative stress [51,52]. Among the 12 evaluated pro-inflammatory mediators, a significant release was observed only for IL-1α, IL-6, IL-8 and TNF-α, consistent with previous observations in epithelial cells derived from other tissues, such as skin keratinocytes [53]. In general, the observed low increase of cytokines release, limited only to few pro-inflammatory cytokines, suggests a general low inflammatory potential. Considering the latter, GO induced 2.6-fold increase of TNF-α release, as compared to negative controls. An increased TNF-α release was also previously observed in A549 cells: as compared to the negative control (TNF-α: 161 pg/mL), 24 h exposure to GO (40 μg/mL) significantly increased TNF-α release to 370 pg/mL (approximately a 2.3-fold increase) [54]. However, the highest effect was observed for rGO, which significantly increased TNF-α release by 3.5-fold, as compared to negative controls. wdGO was less potent, inducing a TNF-α release at levels comparable to that of the negative control. Given the results of the TET assay, it should be noted that these results would not be altered by a probable endotoxin contamination of the materials, which was excluded, and therefore may be an intrinsic property of GO and rGO. An important effect was recorded for IL-1α, whose release was significantly increased only by rGO (7.7-fold, with respect to negative controls). It should be noted that barrier cells, such as bronchial epithelial cells, store significant amounts of IL-1α already at the steady state [55]; therefore, it is also possible that preformed stores of IL-1α in bronchial cells could be released after mechanical damages by rGO. This is in line with the previous hypothesis that the higher detrimental effects of rGO may be due to its sharp structures, affecting cell membranes integrity. 

However, the profile of cytokines released by cells exposed to the three materials suggests a low inflammatory potential, despite some differences that can be evidenced. Indeed, the clustering analysis suggests for GO and rGO a cytokines release pattern with barely more similarities to those of the positive control and reference material than those of negative controls. wdGO appears to be the least inflammogenic material, showing a cytokines release pattern similar to that of negative controls.

Regarding the genotoxic effects, GO, rGO and wdGO were unable to significantly increase the frequency of DNA damage, as compared to the negative control, although a significant linear concentration-dependent response was observed for rGO. Most of the genotoxicity studies assessing GBMs have been performed by the comet assay and showed contradictory results in different cell lines [56]. Regarding lung cells, our results agree with those of Mukherjee and co-workers, who did not observe any induction of DNA damage in human broncho epithelial BEAS-2B cells exposed, for 24 h, to GO or myeloperoxidase-degraded GO sheets (12.5–25 µg/mL) characterized by two lateral dimensions (100 ± 50 nm, and 10 ± 8 µm) [57]. However, in the same cell lines, positive results were recorded for few-layered GO (10 and 50 µg/mL; 24 h exposure), only at the highest concentration [27]. Similarly, GO nanosheets induced DNA alterations in human lung adenocarcinoma A549 cells, although only one concentration (100 µg/mL) was tested, inducing 20% apoptosis [58]. Furthermore, Wang and colleagues observed that the strong genotoxic effects of unfunctionalized GO in human lung fibroblasts, already at 1 µg/mL, were reduced by GO functionalization with different surface groups; lactobionic acid-polyethylene glycol functionalized GO showed no effects at all the concentrations tested (1–100 μg/mL) [24]. Negative results were recorded after treating lung epithelial FE1 cells with few-layered GO or rGO (up to 200 μg/mL) [59]. Interestingly, even if not evaluated in lung cells, but in the human retinal pigment epithelium ARPE-19 cell line, the removal of oxygen-containing functional groups of GO by reduction with Na-citrate induced a significant increase of DNA damage [60], in line with the slightly higher potential of rGO to damage DNA observed in the present work. The increased DNA alterations may be induced by oxidative damage, as suggested by the enhanced ROS production by rGO recorded by Ou and colleagues, as well as in the present study.

Very few studies assessed the ability of GBMs to induce MN [56]. To the best of our knowledge, GO or rGO have not been previously tested in human lung cells, using CBMN assay. As compared to the negative control, no significant differences were found in any of the GBM concentrations evaluated in the present study, although GO induced a significant linear concentration response. Different forms of GO were tested on other cell types [61,62,63], with positive results only being observed in human lymphocytes exposed to GO nanosheets (50 and 100 µg/mL) for 44 h [62]. An increased MN formation was observed exposing 16HBE14o− cells, also used in the present study, to neutral and aminated few-layers graphene (2–100 µg/mL) for 24 h, whereas negative results were recorded for the carboxylated form [64]. Cellular internalization of GBMs was reported in the latter study, confirming the adequacy of the cell line (the same as the one used in the current work) for assessing the genotoxicity of these types of materials. We should emphasise that the cytochemical analysis we performed is unable to strictly demonstrate cellular internalization of GO derivatives, even though some black signals may be ascribable to the presence of GO and rGO depots inside the cells. However, internalization of the studied materials by the cells was demonstrated by confocal microscopy in reflection mode, showing that GO and rGO, but not wdGO, are retained in cells. Cell internalization of GO and different types of rGO was previously noted in various pulmonary cell lines, such as A549 and BEAS-2B cells [25,48,65,66,67]. Overall, these observations support our hypothesis that the lower cytotoxic potential of wdGO with respect to that of GO and rGO could be due to its lower interaction with bronchial cells, possibly leading to a lower mechanical cellular damage.

## 5. Conclusions

Overall, our results indicate, as a main outcome, that rGO is the most cytotoxic material for epithelial bronchial cells among the tested GO derivatives, particularly in terms of cell viability reduction and increased ROS production. The increased cytotoxicity of rGO, as compared to that of GO, could be due to its chemical reduction. Ascorbic acid has been previously employed as reducing reagent for GO, and is considered to be an ideal substitute for hydrazine [68]; however, further studies should be carried out to clarify the impact of the chosen reduction process on rGO toxic potential. As a second result, this study indicates that GO formulated in a stable water dispersion form is highly biocompatible, probably reducing mechano–physical interaction with cell membranes, leading to cell damage. However, it should be considered that the direct comparison of wdGO and powdered GO effects might be biased by the dispersion of the latter in cell media, a step required for cell treatment that could, however, alter some physicochemical properties of the original material. These results acquire a significant importance for physicists, chemists and materials scientists specialized in the field of GBMs, given that dispersed and powdered GO have applications in different technological fields.

## Figures and Tables

**Table 1:** Elemental analysis of GO, rGO and wdGO.

Material	%C	%H	%N	%S	%O
GO	53.3	0.6	0.0	2.5	43.6
rGO	83.3	0.9	0.0	0.0	15.8
wdGO	53.1	1.0	0.0	2.6	43.3

## Data Availability

Data presented in this study are available on request from the corresponding author.

## Supplementary Materials

The following supporting information can be downloaded at: https://www.mdpi.com/article/10.3390/nano13152189/s1, Figure S1: Stability of GBM dispersions.; Figure S2: Kinetic of ROS production in 16HBE14o− cells exposed to GO, rGO or wd(GO); Figure S3: Effects of GO, rGO and wdGO on 16HBE14o− cells viability evaluated by the CellTiter-GloVR Luminescent Cell Viability Assay after 3 h and 24 h exposure.
