# Supplementary material for: Role of Chemical Reduction and Formulation of Graphene Oxide on Its Cytotoxicity towards Human Epithelial Bronchial Cells
Source: Nanomaterials (Basel). 2023 Jul 27;13(15):2189. doi: 10.3390/nano13152189 (PMC10420834; doi:10.3390/nano13152189)
Supplement: Supplementary file 1 [file nanomaterials-13-02189-s001.zip › nanomaterials-2502933-supplementary.pdf]

nanomaterials

Supplementary Material

Role of Chemical Reduction and Formulation of Graphene Oxide on Its Cytotoxicity Towards Human Epithelial Bronchial Cells

Marco Pelin [1,*], Clara Passerino [1], Adriana Rodríguez-Garraus [2], Michela Carlin [1], Silvio Sosa [1], Satu Suhonen [2], Gerard Vales [2], Beatriz Alonso [3], Amaia Zurutuza [3], Julia Catalán [2,4] and Aurelia Tubaro [1]

1 Department of Life Sciences, University of Trieste, Via Fleming 22, 34127 Trieste, Italy; clara.passerino@gmail.com (C.P.); michela.carlin@phd.units.it (M.C.); ssosa@units.it (S.S.); tubaro@units.it (A.T.)
2 Finnish Institute of Occupational Health, Box 40, Työterveyslaitos, 00032 Helsinki, Finland; adriana.rodriguezgarraus@ttl.fi (A.R.-G.); satu.suhonen@ttl.fi (S.S.); gerard.valessegura@ttl.fi (G.V.); julia.catalan@ttl.fi (J.C.)
3 Graphenea S.A., Mikeletegi 83, 20009 San Sebastián, Spain; b.alonso@graphenea.com (B.A.); a.zurutuza@graphenea.com (A.Z.)
4 Department of Anatomy Embryology and Genetics, University of Zaragoza, 50013 Zaragoza, Spain
* Correspondence: mpelin@units.it; Tel.: +39-040-5588620

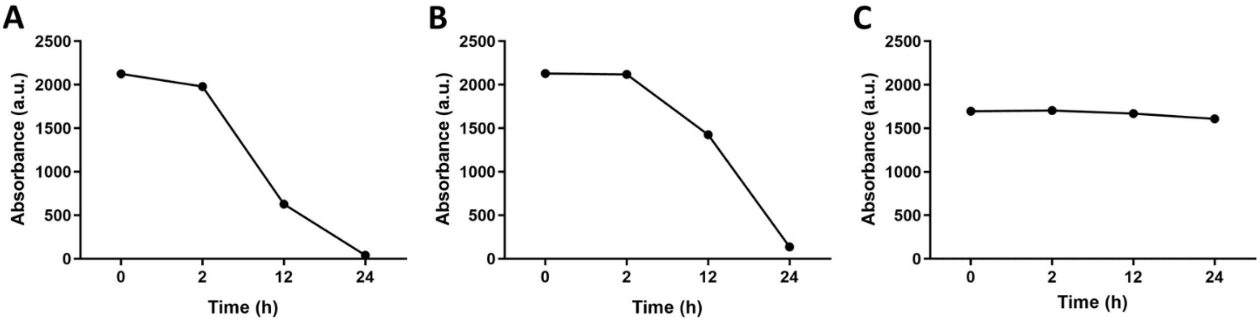

Figure S1. Stability of GBM dispersions. Absorbance values at 660 nm of GO (**A**), rGO (**B**) and wdGO (**C**) dispersion (1 mg/mL) in 0.1% BSA up to 24 h.

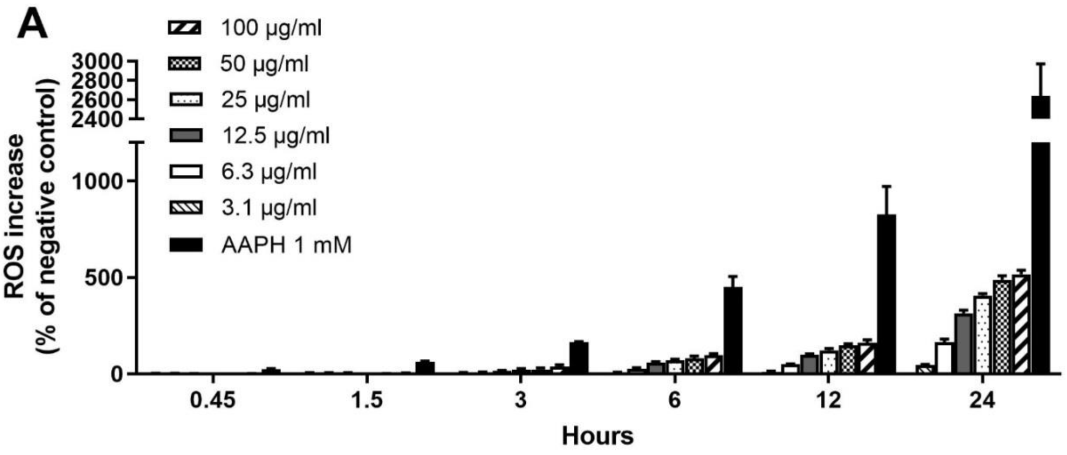

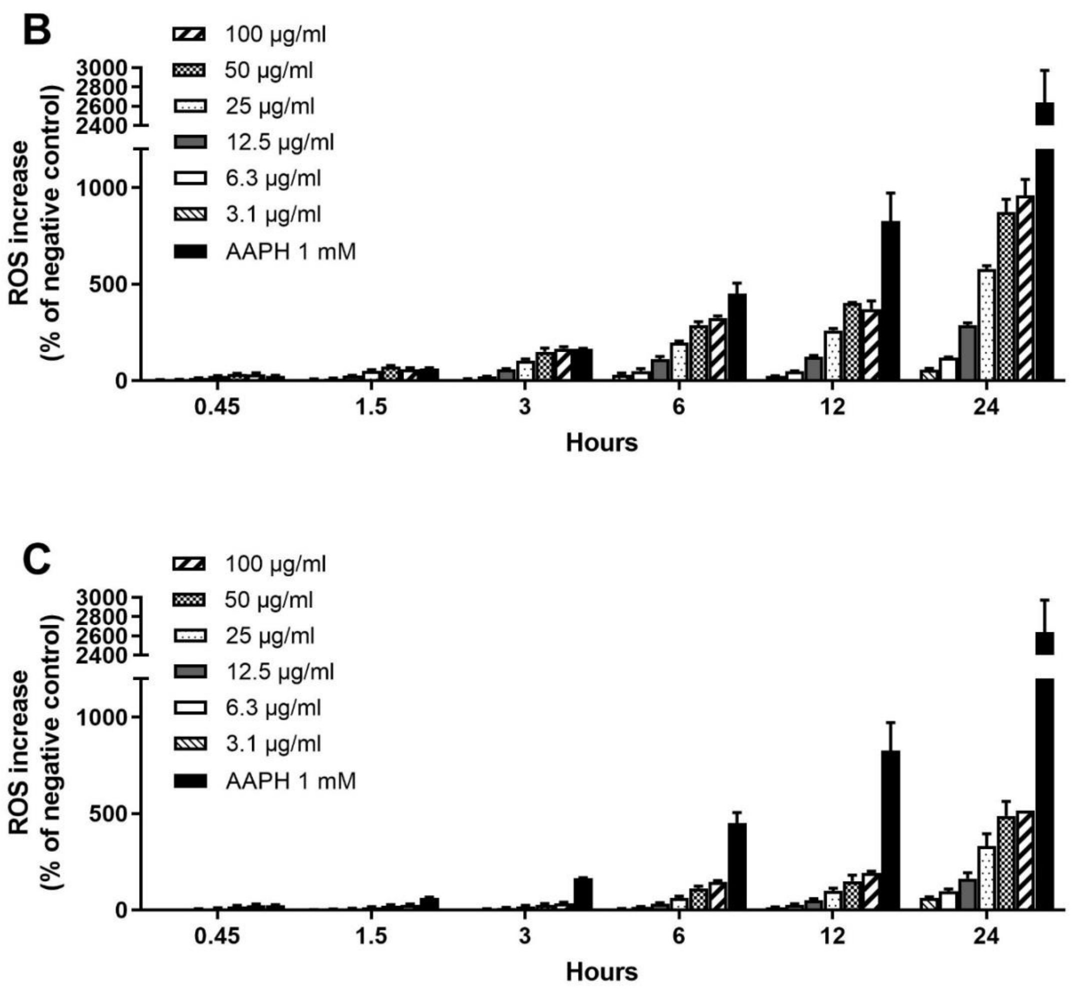

Figure S2. Kinetic of ROS production in 16HBE14o- cells exposed to GO (**A**), rGO (**B**) or wd(GO) (**C**). Cells were exposed to each material for increasing time intervals (from 45 min up to 24 h) and ROS production was evaluated by the DCFDA fluorescence assay. As positive controls, cells were exposed to 1 mM AAPH. Results are reported as % of ROS production with respect to untreated controls and are the mean ± SE of 3 independent experiments.

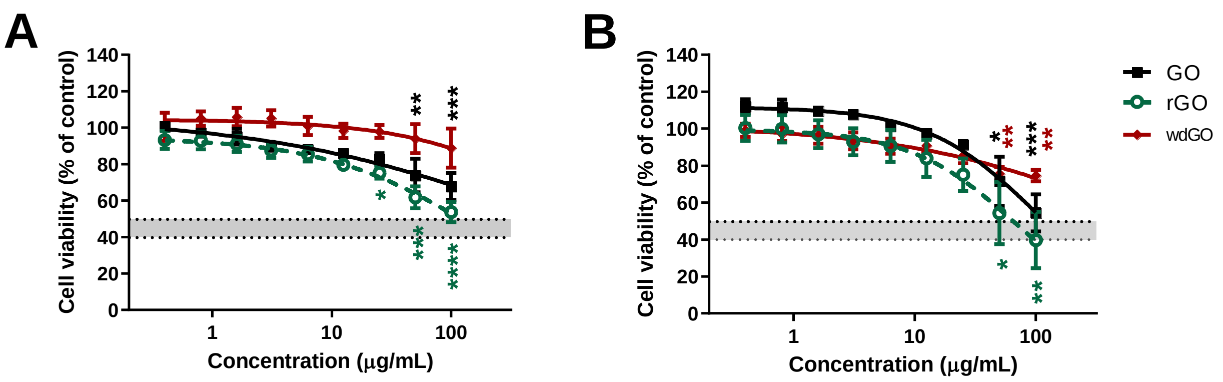

Figure S3. Effects of GO, rGO and wdGO on 16HBE14o− cells viability evaluated by the CellTiter-GloVR Luminescent Cell Viability Assay after 3 h (**A**) and 24 h (**B**) exposure. Data are reported as % of cell viability in cells exposed to GBMs with respect to untreated control cells (negative control) and are the mean ± SE of 3 independent experiments performed in quadruplicate. Statistical differences vs negative control: *, $p < 0.05$; **, $p < 0.01$; ***, $p < 0.001$ (One-way ANOVA and Dunnett's post-

test). The range of 45±5 % cell viability (corresponding to the 55±5% cytotoxicity) is indicated in each graph.
